# Supplementary figures and images for: Nuclear SUN1 stabilizes endothelial cell junctions via microtubules to regulate blood vessel formation
Source: eLife. 2023 Mar 29;12:e83652. doi: 10.7554/eLife.83652 (PMC10059686; doi:10.7554/eLife.83652)

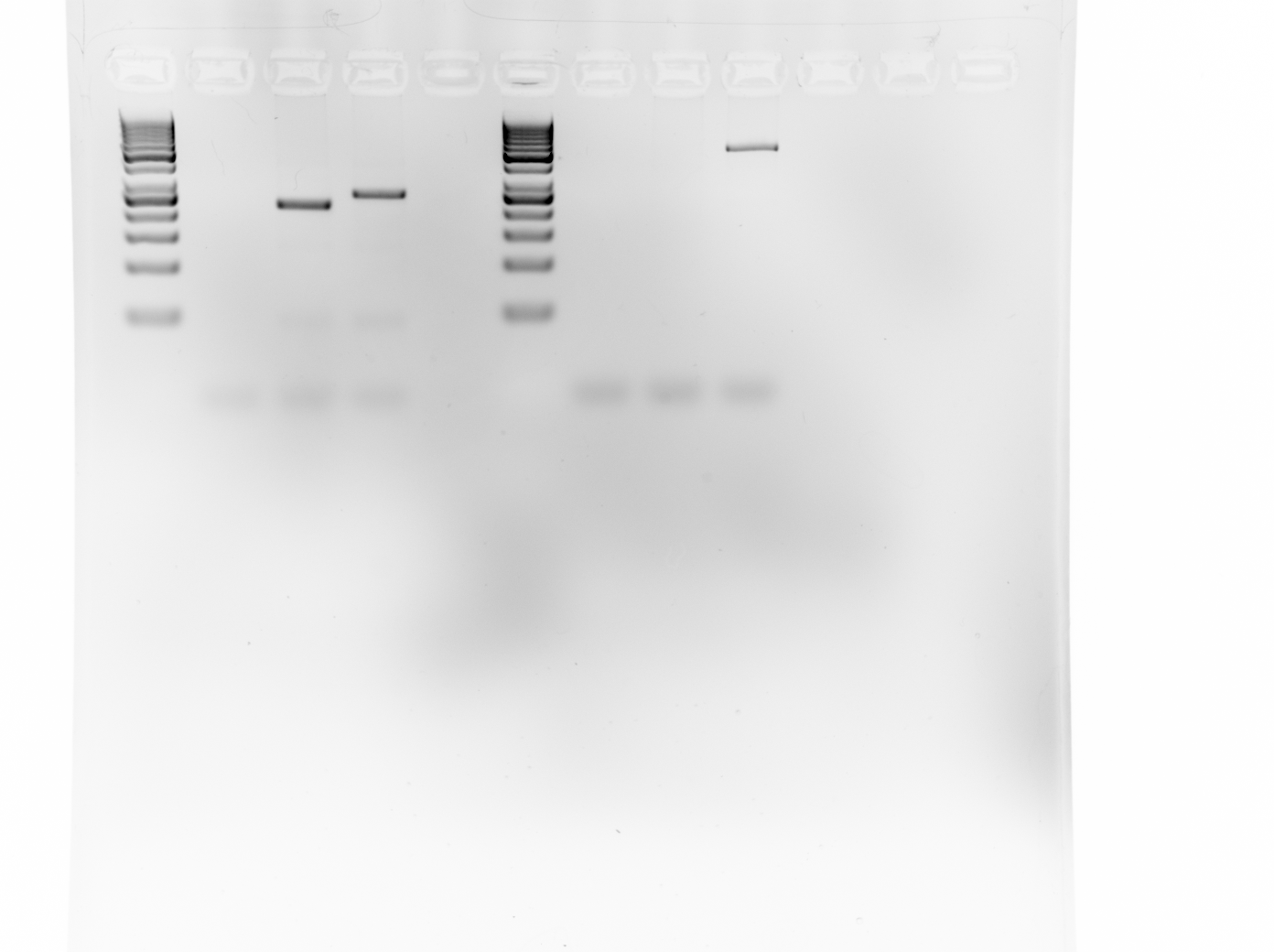

Supplement: Figure 1—figure supplement 1—source data 1. — DNA was extracted from mouse tail cuts. [file elife-83652-fig1-figsupp1-data1.zip › Figure1-figure supplement 1-source data 1/Figure1-figure supplement 1-source data 1.tif]

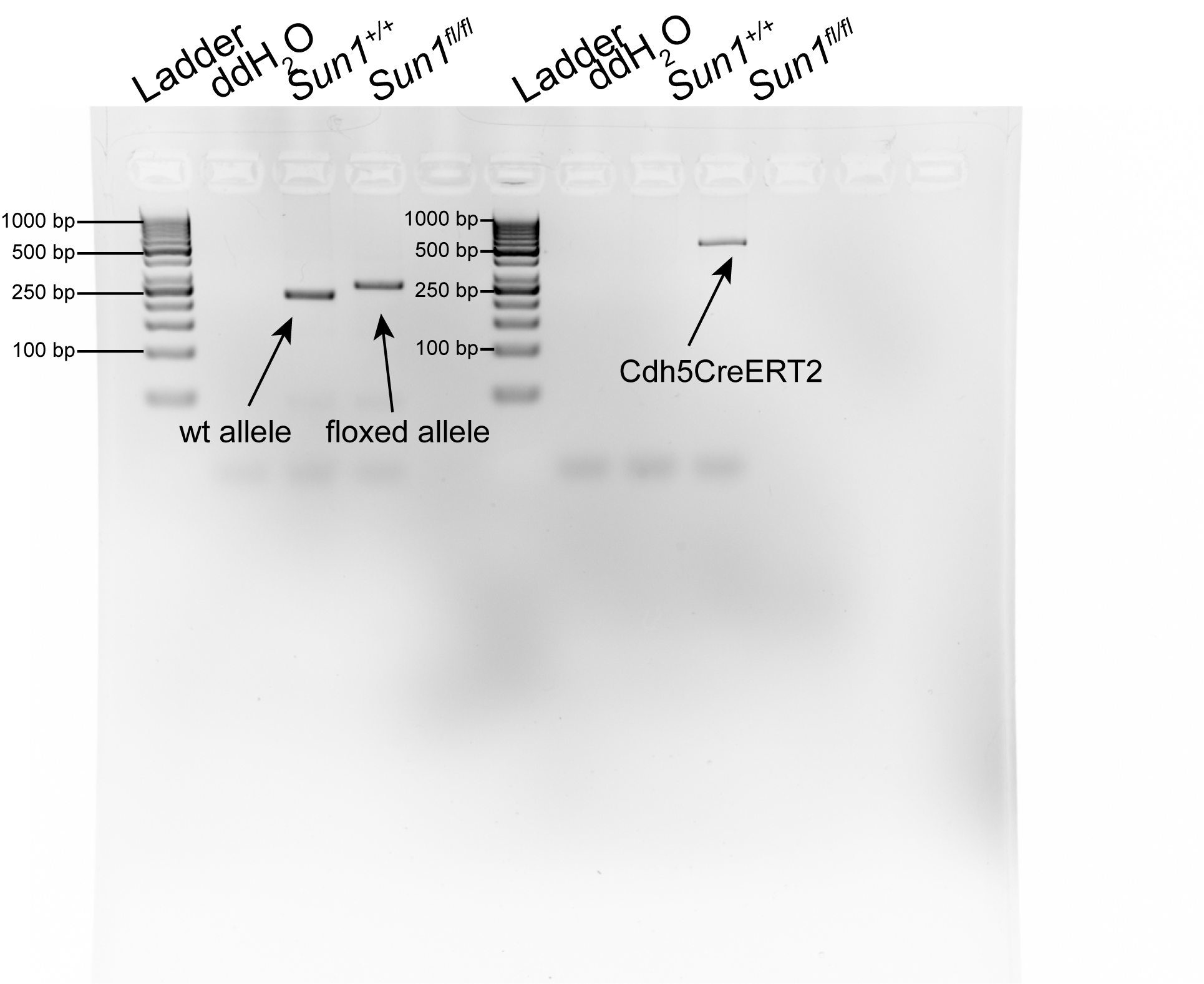

Supplement: Figure 1—figure supplement 1—source data 1. — DNA was extracted from mouse tail cuts. [file elife-83652-fig1-figsupp1-data1.zip › Figure1-figure supplement 1-source data 1/Figure1-figure supplement 1-source data 1_annotated.tif]

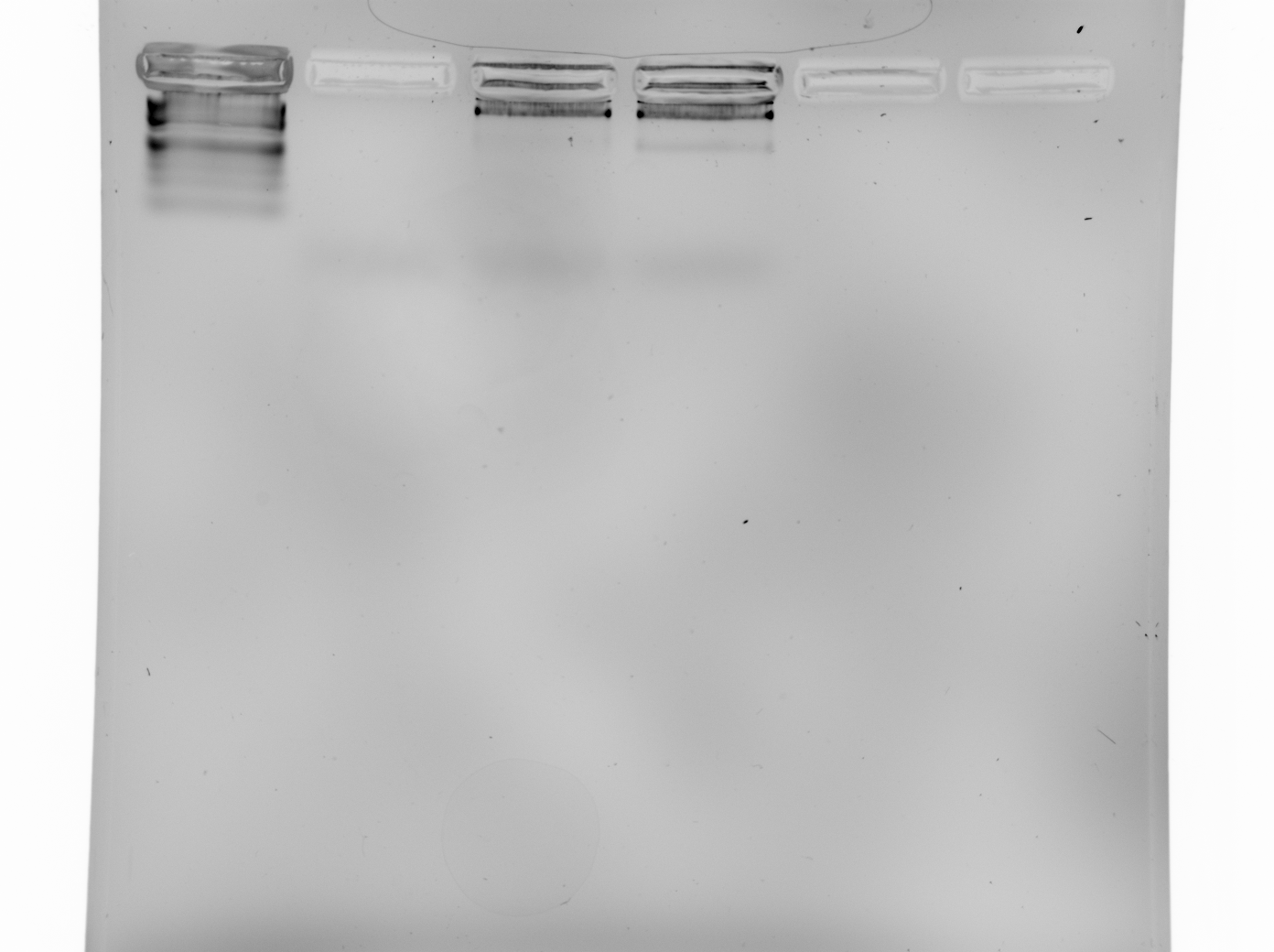

Supplement: Figure 1—figure supplement 1—source data 2. — DNA was extracted from mouse lung tissue. [file elife-83652-fig1-figsupp1-data2.zip › Figure1-figure supplement 1-source data 2/Figure1-figure supplement 1-source data 2.tif]

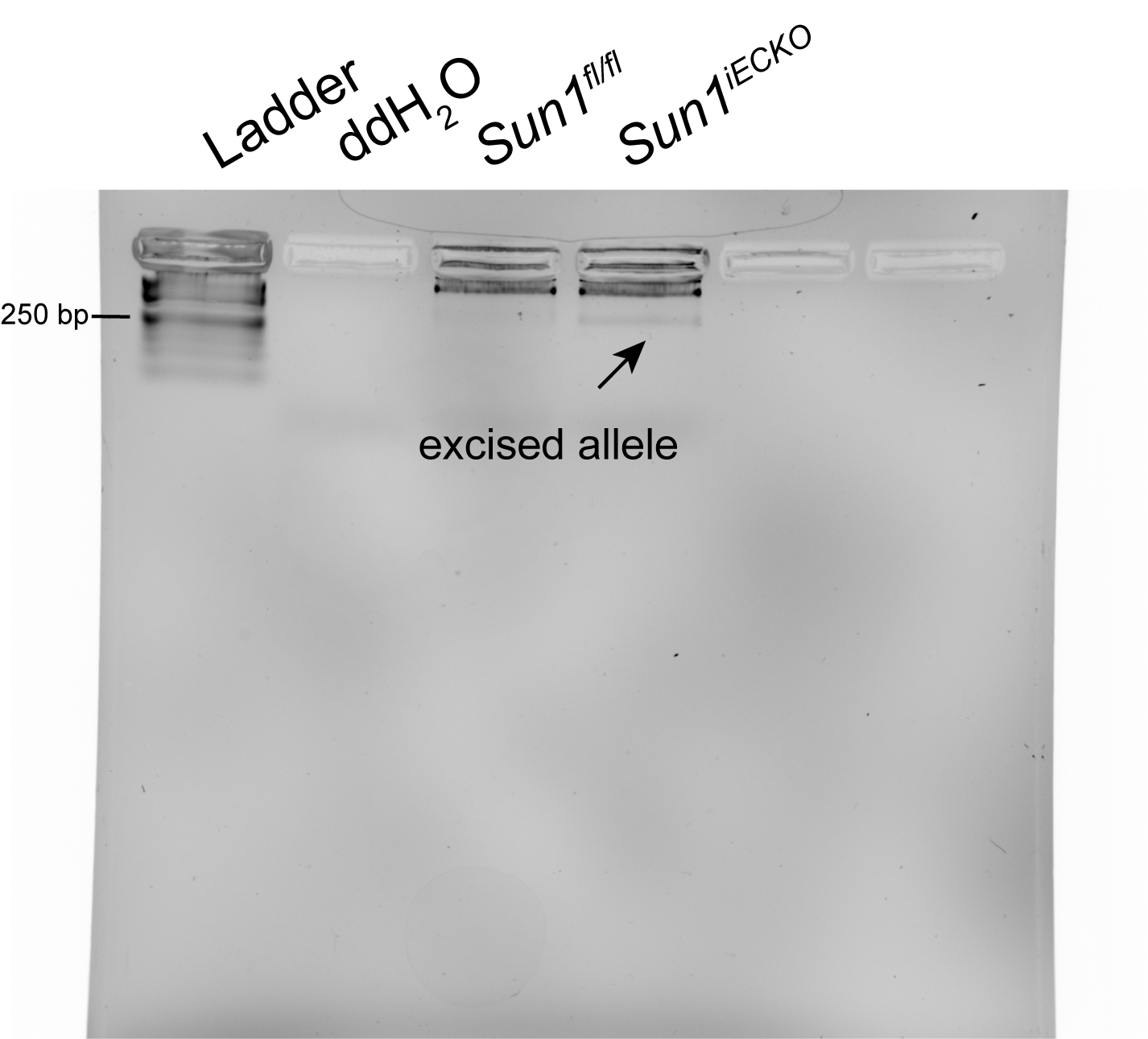

Supplement: Figure 1—figure supplement 1—source data 2. — DNA was extracted from mouse lung tissue. [file elife-83652-fig1-figsupp1-data2.zip › Figure1-figure supplement 1-source data 2/Figure1-figure supplement 1-source data 2_annotated.tif]

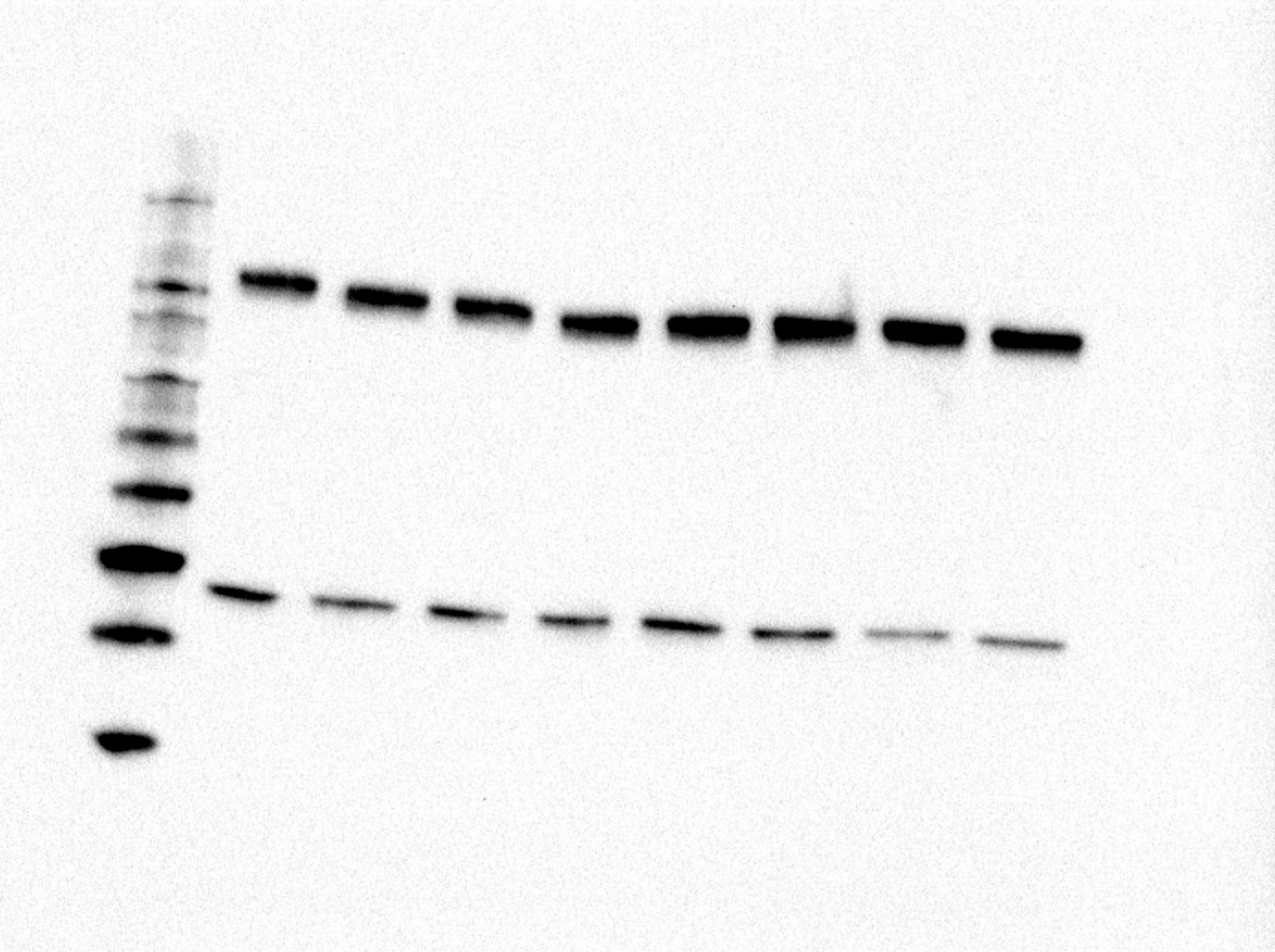

Supplement: Figure 4—figure supplement 1—source data 1. — Protein was extracted from human umbilical vein endothelial cells (HUVEC) with indicated siRNAs. Blot was probed for VE-cadherin (top band) and GAPDH (bottom band) as a loading control. Red box indicates lanes used for Figure 4—figure supplement 1B. [file elife-83652-fig4-figsupp1-data1.zip › Figure 4-supplement 1 source data/Figure4-figure supplement 1-source data 1.tif]

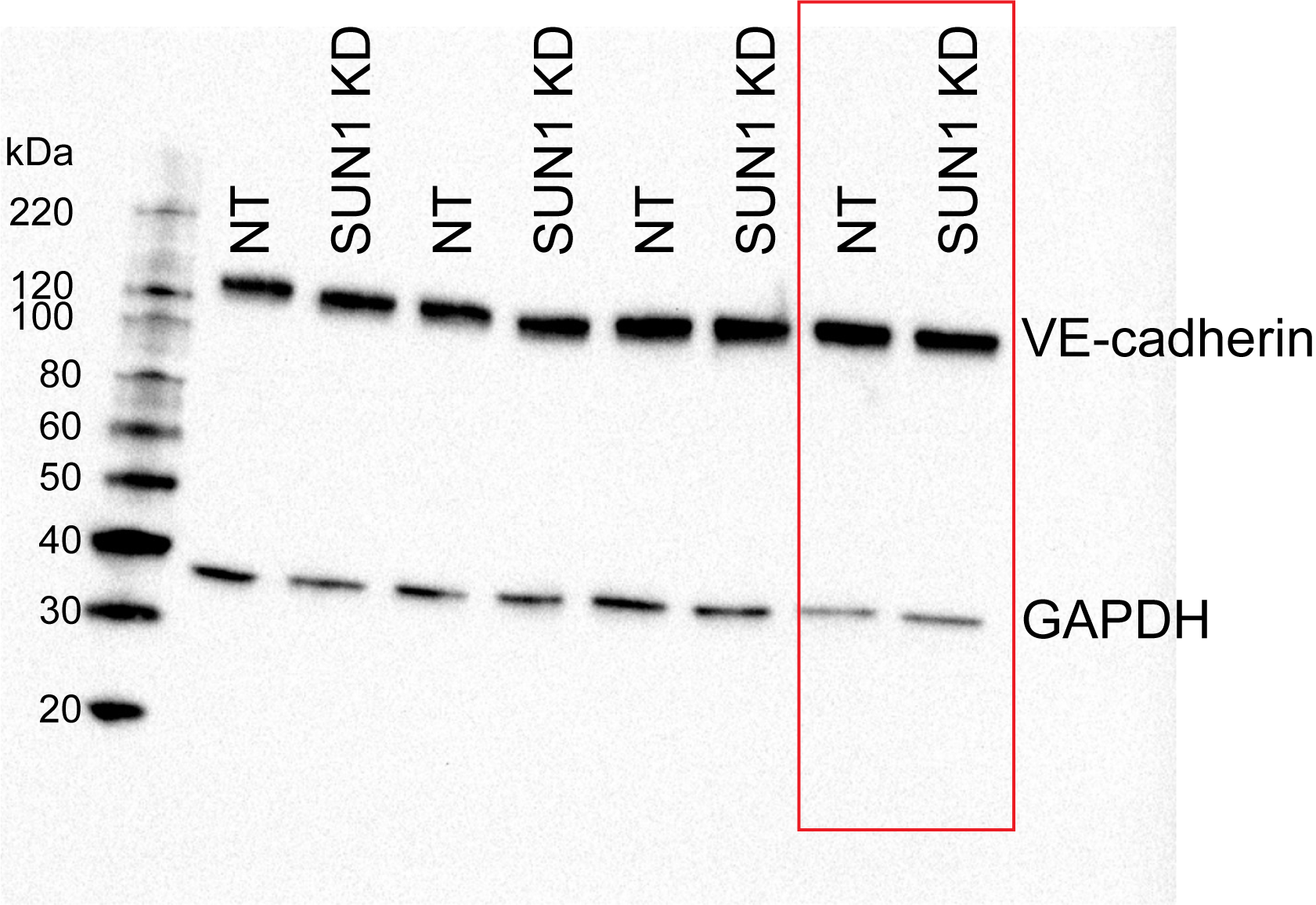

Supplement: Figure 4—figure supplement 1—source data 1. — Protein was extracted from human umbilical vein endothelial cells (HUVEC) with indicated siRNAs. Blot was probed for VE-cadherin (top band) and GAPDH (bottom band) as a loading control. Red box indicates lanes used for Figure 4—figure supplement 1B. [file elife-83652-fig4-figsupp1-data1.zip › Figure 4-supplement 1 source data/Figure4-figure supplement 1-source data 1_annotated.tif]

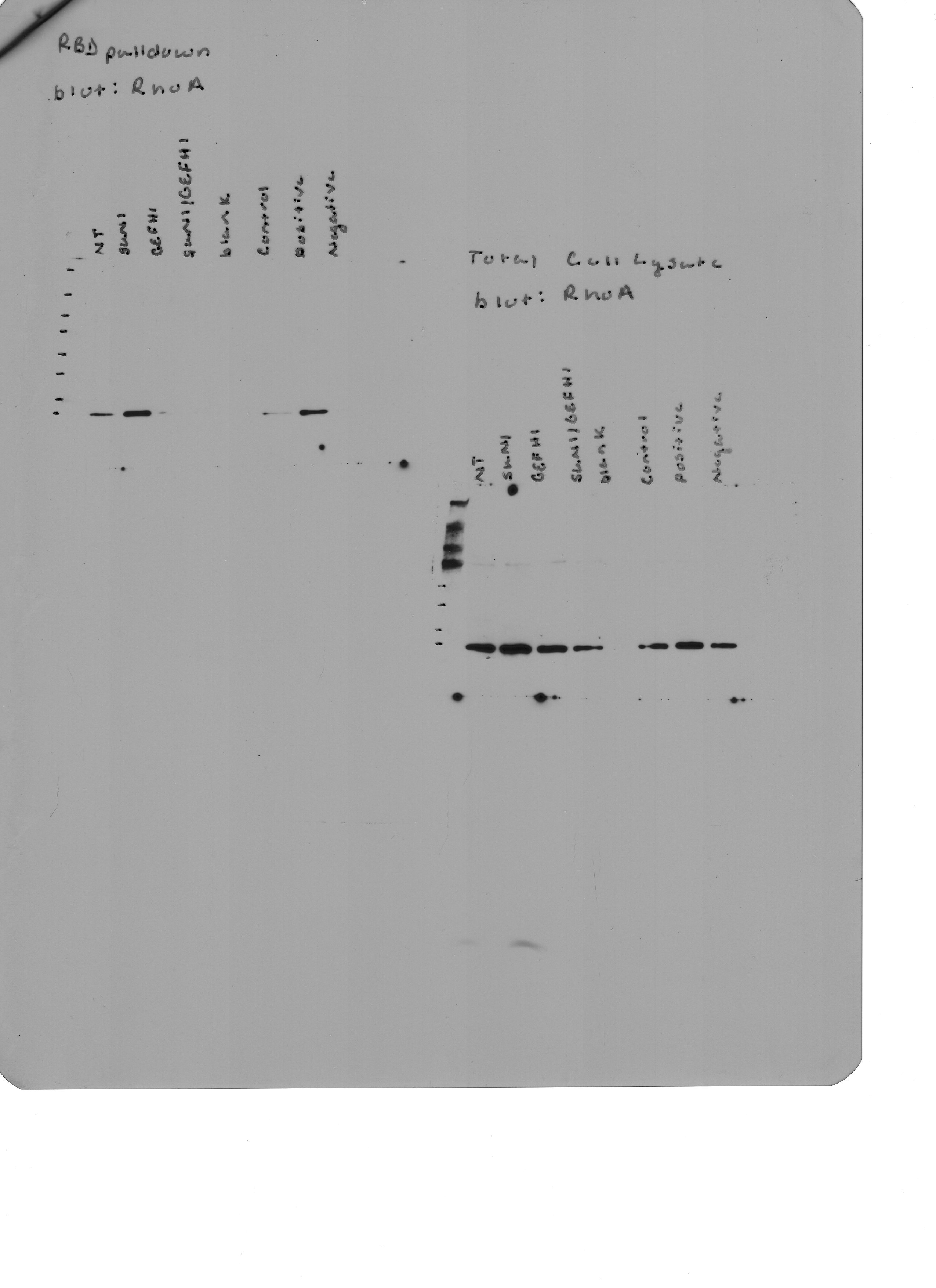

Supplement: Figure 6—figure supplement 2—source data 1. — Protein was extracted from human umbilical vein endothelial cells (HUVEC) with indicated siRNAs and treatments. Blots were probed for RhoA from whole cell lysates (Total RhoA, right blot) or following RBD pulldown (RhoA-GTP, left blot). Thrombin treatment was used as positive control and serum starvation was used as a negative control. Red boxes indicate portions of blots used for Figure 6—figure supplement 2D. [file elife-83652-fig6-figsupp2-data1.zip › Figure 6-supplement 2 source data/Figure6-figure supplement 2-source data 1.png]

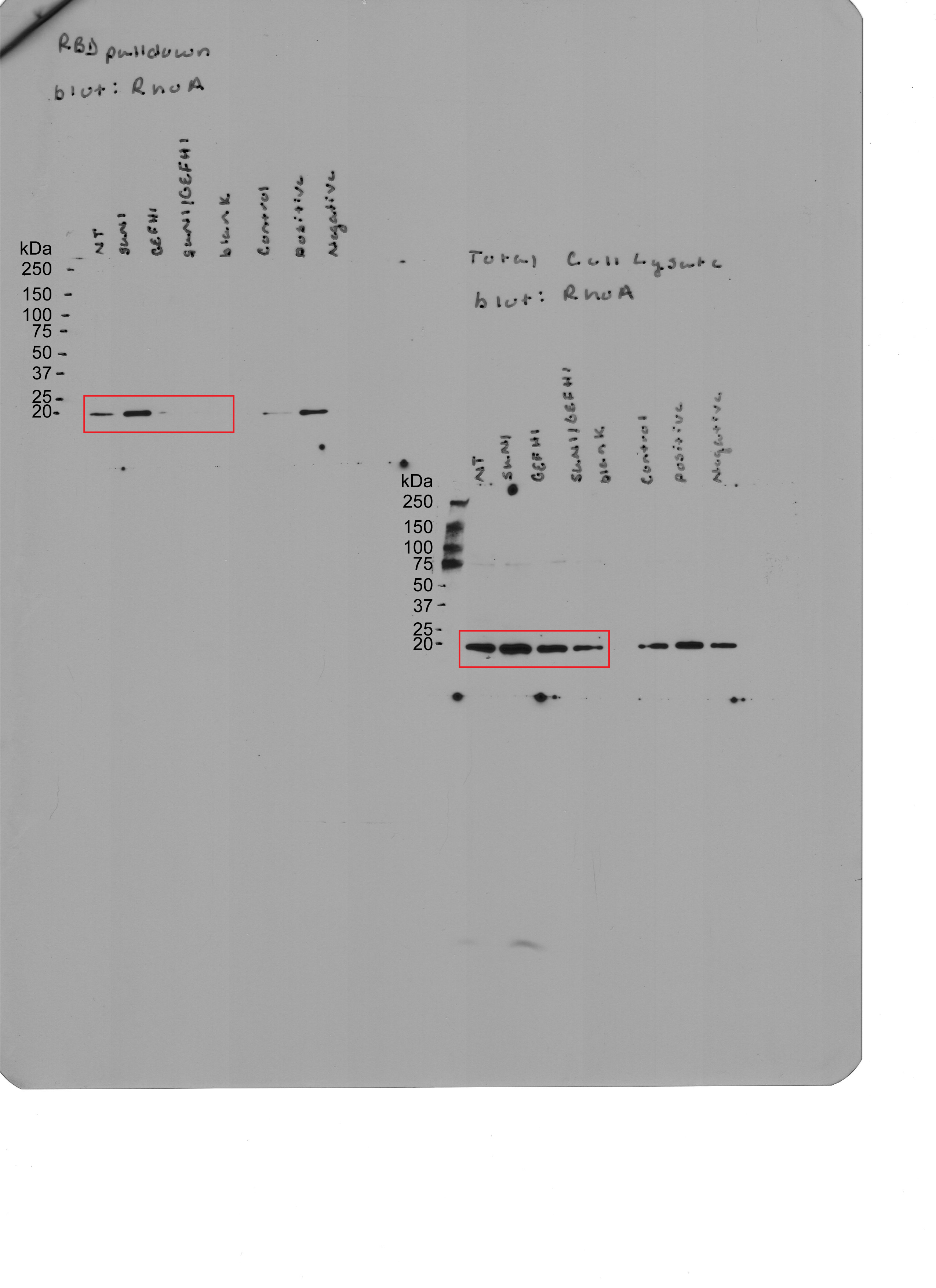

Supplement: Figure 6—figure supplement 2—source data 1. — Protein was extracted from human umbilical vein endothelial cells (HUVEC) with indicated siRNAs and treatments. Blots were probed for RhoA from whole cell lysates (Total RhoA, right blot) or following RBD pulldown (RhoA-GTP, left blot). Thrombin treatment was used as positive control and serum starvation was used as a negative control. Red boxes indicate portions of blots used for Figure 6—figure supplement 2D. [file elife-83652-fig6-figsupp2-data1.zip › Figure 6-supplement 2 source data/Figure6-figure supplement 2-source data 1_annotated.tif]

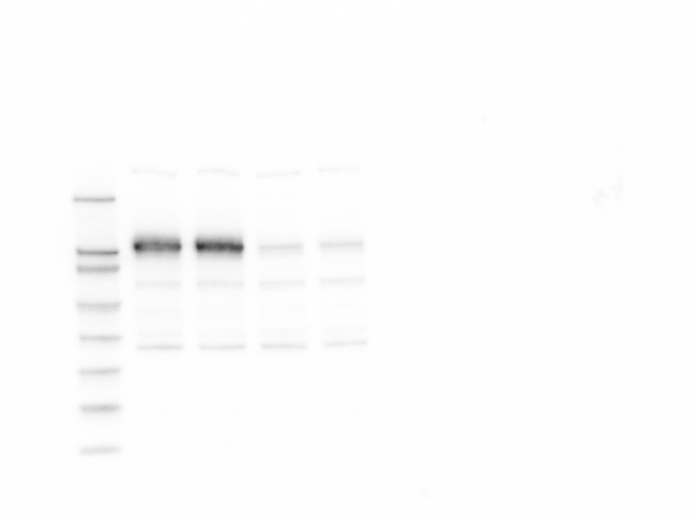

Supplement: Figure 6—figure supplement 3—source data 1. — Protein was extracted from human umbilical vein endothelial cells (HUVEC) with indicated siRNAs. Blot was probed for GEF-H1 (labeled GEF-H1 in source file). Total protein from transfer was used as a loading control (labeled total protein in source file). Red boxes indicate portions of blots used for Figure 6—figure supplement 3A. [file elife-83652-fig6-figsupp3-data1.zip › Figure 6-supplement 3 source data/Figure6-figure supplement 3-source data 1_GEF-H1.tif]

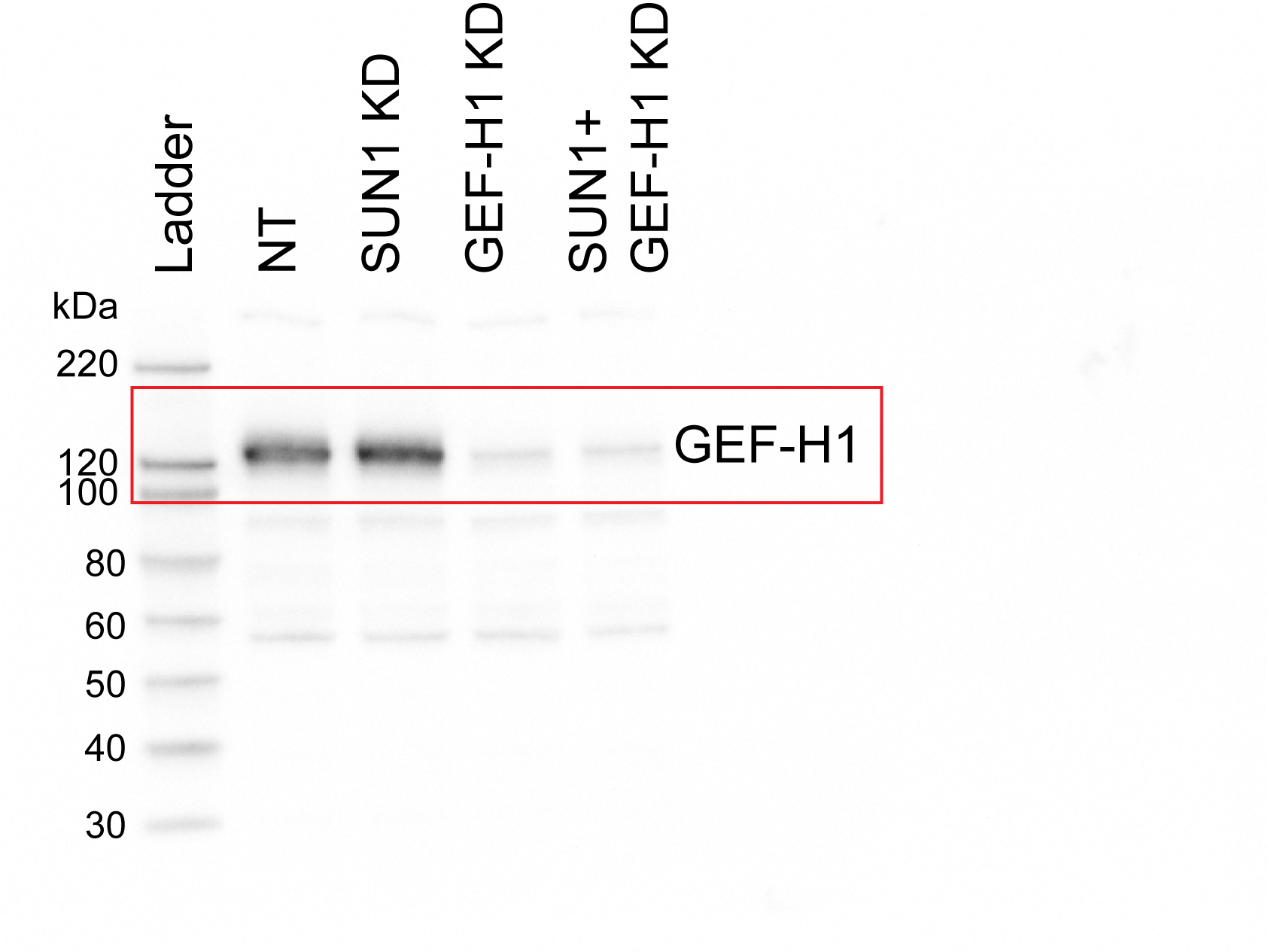

Supplement: Figure 6—figure supplement 3—source data 1. — Protein was extracted from human umbilical vein endothelial cells (HUVEC) with indicated siRNAs. Blot was probed for GEF-H1 (labeled GEF-H1 in source file). Total protein from transfer was used as a loading control (labeled total protein in source file). Red boxes indicate portions of blots used for Figure 6—figure supplement 3A. [file elife-83652-fig6-figsupp3-data1.zip › Figure 6-supplement 3 source data/Figure6-figure supplement 3-source data 1_GEF-H1_annotated.tif]

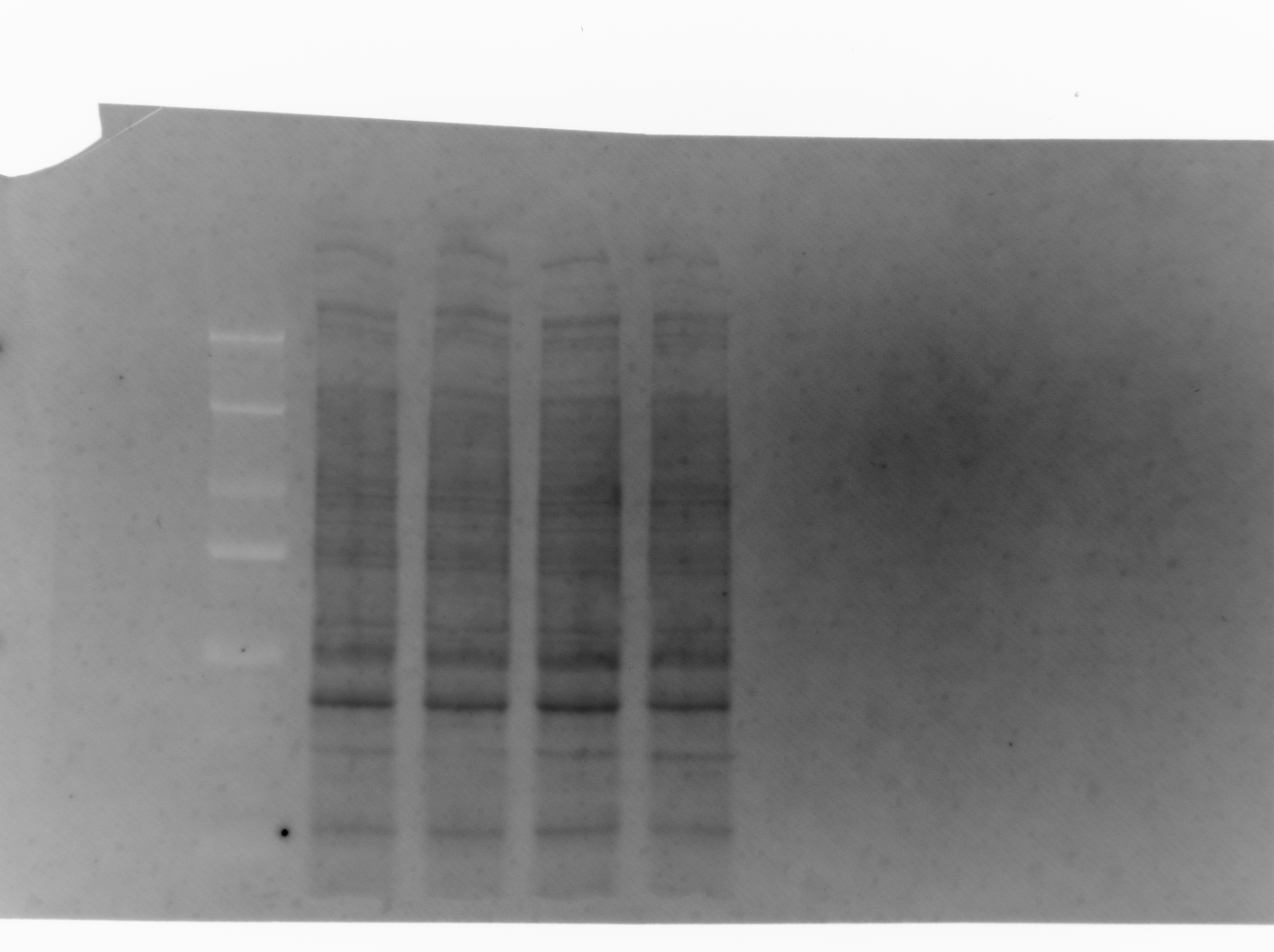

Supplement: Figure 6—figure supplement 3—source data 1. — Protein was extracted from human umbilical vein endothelial cells (HUVEC) with indicated siRNAs. Blot was probed for GEF-H1 (labeled GEF-H1 in source file). Total protein from transfer was used as a loading control (labeled total protein in source file). Red boxes indicate portions of blots used for Figure 6—figure supplement 3A. [file elife-83652-fig6-figsupp3-data1.zip › Figure 6-supplement 3 source data/Figure6-figure supplement 3-source data 1_total protein.tif]

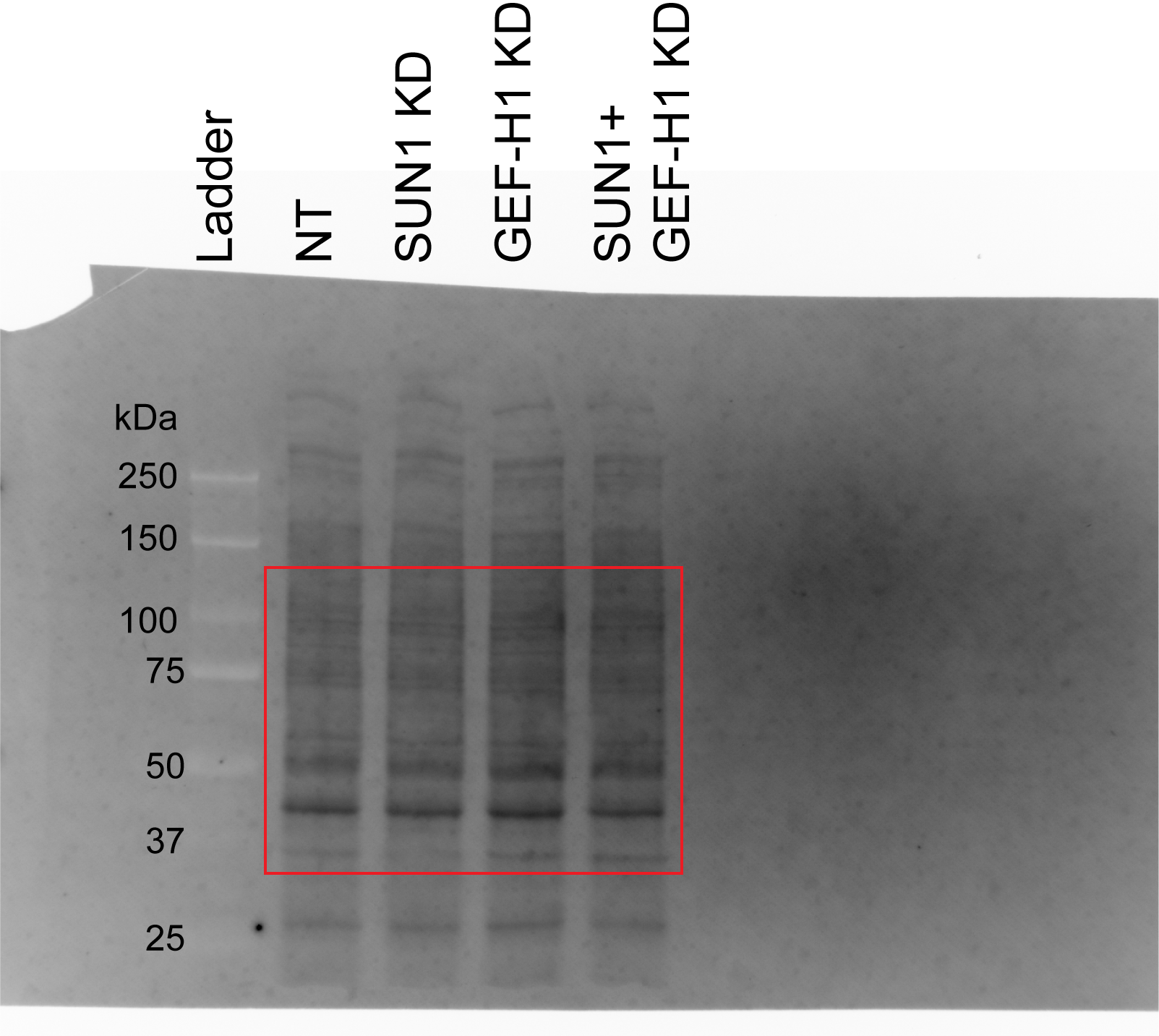

Supplement: Figure 6—figure supplement 3—source data 1. — Protein was extracted from human umbilical vein endothelial cells (HUVEC) with indicated siRNAs. Blot was probed for GEF-H1 (labeled GEF-H1 in source file). Total protein from transfer was used as a loading control (labeled total protein in source file). Red boxes indicate portions of blots used for Figure 6—figure supplement 3A. [file elife-83652-fig6-figsupp3-data1.zip › Figure 6-supplement 3 source data/Figure6-figure supplement 3-source data 1_total protein_annotated.tif]
